# External and Internal Training Loads for Intensive and Extensive Tactical-Conditioning in Soccer Small Sided Games

**DOI:** 10.2478/hukin-2022-0083

**Published:** 2022-09-08

**Authors:** Vinicius Zanetti, Marcelo Saldanha Aoki, Paul S Bradley, Alexandre Moreira

**Affiliations:** 1 Department of Sport, School of Physical Education and Sport, University of São Paulo, São Paulo, Brazil; 2 Red Bull Brazil, São Paulo, Brazil; 3 School of Arts, Sciences, and Humanities, University of São Paulo, Sao Paulo, Brazil; 4 Research Institute of Sport & Exercise Sciences, Liverpool John Moores University, Liverpool, United Kingdom

**Keywords:** internal load, external load, game-based training, elite athletes

## Abstract

This study aimed to investigate the external (ETLs; 15-Hz GPS unit coupled with a 100 Hz tri-axial accelerometer) and internal training loads (ITLs; session-RPE method) of 18 elite U20 soccer players (19 ± 1.0 years, 178 ± 8 cm, 71 ± 7 kg) undertaking a tactical-conditioning training program with special reference to small-sided games (SSGs). The SSGs used in this program were either extensive (SSG-extensive) or intensive (SSG-intensive) training sessions, that were alternated within the assessed weeks. Tactical principles of the game influenced the aim of the technical-tactical content. Total distance (TDR; ES: 1.17), and a very high-speed running distance (HSR; ES: 0.96) were greater in SSG-extensive vs. SSG-intensive. However, no meaningful difference was found for accelerations (ACC; ES: 0.12) and decelerations (DEC; ES: 0.08). However, a higher perceived intensity (session-RPE; ES: 0.62) and greater ITLs (ES: 0.27) were found for SSG-intensive. These findings suggest that coaches should prescribe SSG training sessions not only considering the usual variables (rules, player numbers, etc.). The current data indicate that the tactical objective of SSGs in relation to exercise bout duration and rest intervals should be considered, while implementing a multi-dimensional training monitoring program during SSG tactical-conditioning training sessions, integrating ETL and ITL variables to gain a better understanding of training responses.

## Introduction

In the applied setting, soccer coaches typically use various small-sided-games (SSGs) as the main training method in order to alternate the stimulus within a week (Ade et al., 2014; Aguiar et al., 2012). The rationale of using SSGs as the predominant training method is that many variables can be manipulated to provide a stimulus aligned with the objective of the session. For instance, the pitch size, the number of players, and rules are different to those for full size competitive matches (Sangnier et al., 2019). SSGs not only mimic some of the game demands, but they can actually overload players physically while still developing technical and decision-making skills (Ade et al., 2014; Aguiar et al., 2012; Martín-García et al., 2018). Despite these assumptions and the adoption of SSGs as one of the main training methods used by coaches for soccer training, aimed to develop technical and tactical abilities (Halouani et al., 2014), limited data exist regarding the loading patterns of different SSGs using a tactical-conditioning training program. The development of tactical expertise is imperative for athletic performance in team sports, which has been defined as a successful interaction in decision making skills in order to respond to tactical requirements of the game (Gréhaigne and Godbout, 1995). In soccer, some propose both attacking and defensive operational principles that oppose each other (Teoldo et al., 2009), and these should be considered during training practice to improve tactical knowledge (Halouani et al., 2014). Using these operational principles to prescribe SSGs might increase the likelihood of improving tactical knowledge.

Despite the importance of using tactical principles to guide SSG training sessions, limited data exist regarding the external and internal loading patterns of different SSGs mainly oriented through a tactical-conditioning training program. These SSGs could be planned to tax tactical-technical elements while simultaneously developing physical qualities. SSG formats could be therefore alternated to target and develop tactical elements based on tactical principles of soccer (Teoldo et al., 2009). Indeed, incorporating the terms “intensive” and “extensive” SSGs, referring to the planned SSG tactical-conditioning format (i.e., tactical aims, the number of players, duration of the exercise bouts, the playing area) could aid in the training prescription while allowing examination of the training load patterns of SSGs. Therefore, this study examined external and internal training load patterns of elite players assessing the impact of two distinct SSG tactical-conditioning sessions (extensive and intensive SSGs).

## Methods

### Participants

Thirty outfield players representative of an elite U20 soccer team competing in both the Brazilian first division and international competitions initially volunteered to participate in this study. Twelve players were excluded because of injury or participating in less than 90% of the SSG training sessions. Therefore, data from 18 players were retained for analysis (age: 19 ± 1.0 years, stature: 178 ± 8 cm, body mass: 71 ± 7 kg; Yo-Yo IR1 performance: 1888 ± 366 m). The assessed team was classed as elite as all players were professional and full time. The study was carried out in accordance with the Code of Ethics of the World Medical Association (Declaration of Helsinki) for experiments involving humans, and all procedures received the University Ethics Committee’s approval. Players underwent a thorough medical assessment to verify their health status prior to participation and were free from illness or injury at the time of the study. Players participated on average in 10–12 hr of soccer training and competitive play per week.

#### Design and procedures

Data were collected in the 6-week training period prior to an U20’s national tournament. This pre-competitive training period was initiated after one week of full recovery, as the team was already in the in-season period, participating in the state competition. This tournament is a major national competition for this age-category. The assessed team advanced to the playoff rounds of the tournament. During this period, coaches used a periodization approach applied to tactical-conditioning SSGs. These were developed to tax tactical-technical elements while simultaneously developing physical qualities. SSG formats were alternated to target and develop tactical elements based on tactical principles of soccer (Teoldo et al., 2009), alternating extensive and intensive planned SSG training sessions. Extensive SSG training sessions were prescribed using large and voluminous exercise bouts, while the prescription of intensive SSG training sessions was based on short exercise bouts interspersed with rest intervals. These sessions were alternated within the week. The tactical principles that guided the content of SSGs were adapted from previous literature (Teoldo et al., 2009) and used to design the aim of the SSGs. All players were largely familiarized with the SSG formats used in the training sessions, as they were habitually used in training in order to develop both physical and technical-tactical attributes. Coaches usually already divided the teams according to the playing position and the technical and tactical level and were asked to maintain this procedure during the investigation period. Such players’ division was used in previous investigations (Aguiar et al., 2013; Moreira et al., 2016, 2017; Rodrigues Lopes et al., 2022). Table 1 contextualizes the aims and SSG formats during this 6-week training period, while Table 2 details how extensive and intensive SSGs were alternated during each week. A total of 15 extensive and 15 intensive SSG training sessions were evaluated. The total duration of the main part (phase) of the SSG training sessions ranged from 45 to 70 min (excluding warm-up and cool-down phases). All the assessed SSGs were played at the same time of the day (between 2:30 and 3:00 pm). Players were allowed to drink water *ad libitum*, but not sports drinks (i.e., isotonic consumption, before or during the SSGs). Players abstained from consuming food and caffeine-containing products for at least two hours before the beginning of the SSG. All players had their lunch at the teams' training facility (habitually between 12:00 and 12:30 pm). Mean data of all extensive and intensive SSG training sessions, for the dependent variables, were retained for analysis. External training loads (ETLs) were monitored during all sessions using a 15-Hz GPS unit coupled with a 100 Hz tri-axial accelerometer. The session-RPE and internal training loads (ITLs) were also determined in all SSG sessions.

**Table 1:** Contextualisation of the 6-week training schedule prior to the tournament.

SSG-Extensive Training Sessions

Offensive and defensive coverage and width and length training aimed at transitions (attack-defence and defence-attack) and pressure match-play situations

Typical extensive training session 1, with emphasis on large and voluminous bouts duration

SSG - Transition 5 vs. 0 (width × depth, 40 × 55 m, 20 min, alternating group of players).
SSG – 7 vs. 7 vs. 7 + 3 goalkeepers (width x depth, 40 x 55 m, 1 x 25 min)

Typical extensive training session 2, with emphasis on large bouts duration interspersed with rest intervals

SSG – 7 vs. 7 vs. 7 /+ 3 goalkeepers (GK) (width × depth, 30 × 25 m, 3 x 3 with 2 min rest intervals)
SSG - 7 vs. 5 + 2/ + 2 GK (width × depth, 40 × 55 m, 6 x 3 with 2 min rest intervals)

Typical extensive training session 3, aimed at offensive and defensive unit training (attack) and delay (defence) training

SSG – 5 vs. 4 (attack vs defence) / + 1 GK (width × depth, 30 × 25 m, 1 x 20 min)
SSG – 10 vs. 9 (attack vs defence) / + 2 GK (width × depth, 40 × 55 m, 1 x 30 min)

SSG-Intensive Training Sessions
Penetration and emphasis on goal attempts from short-distance, width and length (attack) and delay (defence) training, and offensive and defensive coverage and width and length training aimed at transitions (attack-defence and defence-attack) and pressure match-play situations

Typical intensive training session 1

SSG – 3 vs. 1 (width × depth, 15 × 15 m, 2 x 30 s with 30 s rest intervals)
SSG – 5 vs. 5 vs. 5 / + 2 GK (width × depth, 30 × 25 m, 5 x 3 min, with 90 s rest intervals)
SSG – 5 vs. 5 / + 1 GK (width × depth, 30 × 25 m, 6 x 3 min, with 2 min rest intervals)

Typical intensive training session 2

SSG – 3 vs. 1 (width × depth, 15 × 15 m, 7 “blocks” x [2 x 30 s with 30 s rest intervals] with 3 min rest between “blocks”)
SSG – 3 vs. 3 + 2 (width × depth, 30 × 25 m, 5 x 3 min, with 90 s rest intervals) - increased emphasis on transition
SSG – 3 vs. 3 (width × depth, 30 × 25 m, 3 x 8 min with 2 min rest intervals) - increased emphasis on goal attempts from long-distance with 1 GK.

Typical intensive training session 3

SSG – 3 vs. 1 (width × depth, 15 × 15 m, 2 x 30 s with 30 s rest intervals)
SSG – 3 vs. 3 + 2 (width × depth, 30 × 25 m, 5 x 3 min, with 90 s rest intervals) - increased emphasis on transition
SSG – 3 vs. 3 (width × depth, 30 × 25 m, 3 x 8 min with 2 min rest intervals) - increased emphasis on goal attempts from short-distance, with 4 GK.

**Table 2:** Typical daily training content during the 6-week assessed period

	Weeks 1, 3, 5	Weeks 2, 4, 6

	SSG - Transition 5 vs. 0 (width × depth, 40 × 55 m, 20 min, alternating group of players).	SSG – 5 vs. 5 vs. 5 / + 2 GK (width × depth, 30 × 25 m, 5 x 3 min, with 90 s rest intervals)
Monday	SSG – 7 vs. 7 vs. 7 + 3 goalkeepers (width x depth, 40 x 55 m, 1 x 25 min)	SSG – 5 vs. 5 / + 1 GK (width × depth, 30 × 25 m, 6 x 3 min, with 2 min rest intervals)

	SSG – 3 vs. 1 (width × depth, 15 × 15 m, 7 “blocks” x [2 x 30 s with 30 s rest intervals] with 3min rest between “blocks”)	SSG – 5 vs. 4 (attack vs. defence) / + 1 GK (width × depth, 30 × 25 m, 1 x 20 min)
		
Tuesday	SSG – 3 vs. 3 + 2 (width × depth, 30 × 25 m, 5 x 3 min, with 90 s rest intervals) - increased emphasis on transition	SSG – 10 vs. 9 (attack vs. defence) / + 2 GK (width × depth, 40 × 55 m, 1 x 30 min)

	SSG – 5 vs. 4 (attack vs. defence) / + 1 GK (width × depth, 30 × 25 m, 1 x 20 min)	SSG – 3 vs. 3 (width × depth, 30 × 25 m, 3 x 8 min with 2 min rest intervals) - increased emphasis on goal attempts from long-distance, with 1 GK.
	SSG – 10 vs. 9 (attack vs. defence) / + 2 GK (width × depth, 40 × 55 m, 1 x 30 min)	
Wednesday		SSG – 3 vs. 3 (width × depth, 30 × 25 m, 3 x 8 min with 2 min rest intervals) - increased emphasis on goal attempts from short-distance, with 4 GK.

	SSG – 3 vs. 3 + 2 (width × depth, 30 × 25 m, 5 x 3 min, with 90 s rest intervals) - increased emphasis on transition	SSG - Transition 5 vs. 0 (width × depth, 40 × 55 m, 20 min, alternating group of players).
Thursday	SSG – 3 vs. 3 (width × depth, 30 × 25 m, 3 x 8 min with 2 min rest) - increased emphasis on goal attempts from long-distance, with 4 GK.	SSG – 7 vs. 7 vs. 7 + 3 goalkeepers (width x depth, 40 x 55 m, 1 x 25 min)

	SSG - 7 vs. 5 + 2/ + 2 GK (width × depth, 40 × 55 m, 6 x 3 with 2 min rest intervals)	SSG – 3 vs. 1 (width × depth, 15 × 15 m, 7 “blocks” x [2 sets x 30 s with 30 s rest intervals] with 3 min rest between “blocks”)
	SSG – 7 vs. 7 vs. 7 /+ 3 goalkeepers (GK) (width × depth, 30 × 25 m, 3 x 3 with 2 min rest intervals)	
Friday		SSG – 3 vs. 3 + 2 (width × depth, 30 × 25 m, 5 x 3 min, with 90 s rest intervals) - increased emphasis on transition

Saturday	Simulated Match play or friendly match	Simulated Match play or Friendly Match

Sunday	Off	Off

All training sessions were preceded by a technical warm-up (passing, ~10 min). A 10 min cool down was conducted after each training session

### Measures

#### External Load Measures

Each player wore a 15-Hz GPS unit coupled with a 100 Hz tri-axial accelerometer (SPI Elite, GPSports, Canberra, Australia). Each unit was harnessed between the shoulder blades and anchored using an undergarment to minimize movement. These units provide more valid and reliable measures of total and high-speed distance compared to 1- and 5-Hz units (Johnston et al., 2014). Physical performance variables included the total distance running covered (TDR), distance covered at very high-speed running (HSR; > 20 km·h^-1^) as well as accelerations (ACCs) and decelerations (DECs) (>2.0 m·s^-2^ and -2.0 m·s^-2^, respectively) as used previously (Zanetti et al., 2021). All variables were normalized per minute of playing time.

#### Intensity and Internal Training Loads of SSG training sessions

To determine the intensity and the internal training load (ITL) for each SSG training session, the players’ session-RPE was recorded ~30 min after each session using the adapted Borg 10-point scale (CR-10 scale) (Foster, 1998). The session-RPE was quantified by asking each player ‘*how intense was your session?’*. The indicated value in the CR-10 scale was used as a perceived intensity indicator of the session. Players were already familiarized with the CR-10 scale before the study. Moreover, the daily ITL was calculated by multiplying the session-RPE by session duration (Foster, 1998). The ITL was determined as the product of the value indicated by the player in the CR-10 scale (session-RPE) and the duration of the entire SSG training session. The validity of the session-RPE method for monitoring loads in soccer players has previously been demonstrated (Impellizzeri et al., 2004).

### Statistical Analysis

Values are presented as means and standard deviations. Effect sizes (ESs) were calculated as the standardized mean difference to determine the meaningfulness of the difference between intensive and extensive SSGs, corrected for bias using Hedge’s formula (Hedge’s g uses pooled weighted standard deviations), and presented with 90% Confidence Limits (CL) (Batterham and Hopkins, 2006). ESs with values of 0.2, 0.5, and 0.8 were considered small, medium, and large differences, respectively (Cohen, 1988). Data were analysed using Microsoft Excel (Microsoft™, USA).

## Results

**Figure 1:**
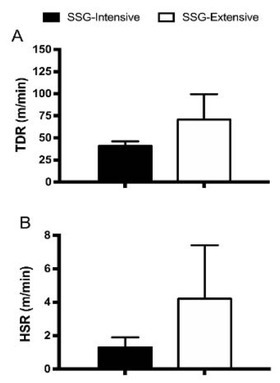
Total distance running (TDR; Figure 1A) and high-speed running (HSR; Figure 1B) for extensive and intensive SSG sessions (mean and SD).

Figure 1 presents the data from total distance (TDR) and high-speed running distance (HSR) for SSG training sessions. A greater TDR (ES: 1.17; CL: 1.51 to 0.84) and a greater HSR (ES: 0.96; CL: 1.29 to 0.63) were covered in SSG-extensive vs. SSG-intensive. The mean values for the within player coefficient of variation (CV) for the relative TDR and HSR for the evaluated SSGs were 7% and 24%, respectively.

**Figure 2:**
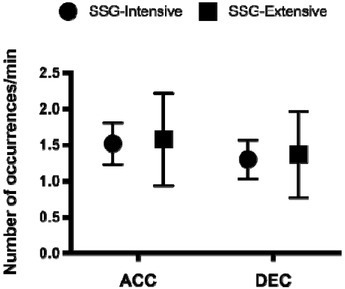
Accelerations (ACC) and decelerations (DEC) for extensive and intensive SSG sessions (mean and SD).

However, no meaningful difference was found for accelerations (Extensive vs. Intensive SSG) (ACCs; ES: -0.12; CL: -0.43 to 0.19) and decelerations (DECs; ES: 0.08; CL: 0.39 to -0.23) (Figure 2). The mean values for the within player CV for relative ACCs and DECs for SSGs were 13% and 18%, respectively.

**Figure 3:**
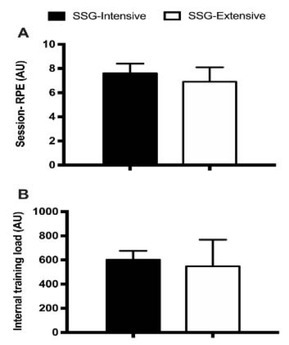
Session-RPE and Internal Training Load (ITL) for extensive and intensive SSG sessions (mean and SD).

A higher perceived intensity (session-RPE; ES: 0.62; CL: 0.30 to 0.94) and a greater ITL (ES: 0.27; CL: -0.04 to 0.59) were observed for SSG-intensive (Figure 3).

**Figure 4:**
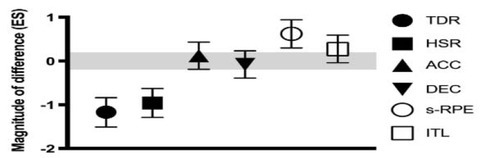
Magnitude of differences between SSG-intensive and SSG-extensive training loads. TDR = total distance running; HSR = high-speed running; ACC = accelerations; DEC = decelerations; ITL = internal training load. Negative ES = lower values for SSG-intensive; positive ES = higher values for SSG-intensive. Grey bar denotes an effect size (ES) > 0.20.

Figure 4 summarizes the magnitude of differences between SSG-intensive and SSG-extensive training loads. A negative effect size (ES) indicates a lower value for SSG-intensive and a positive ES indicate a higher value for SSG-intensive.

## Discussion

This was the first study to examine training load patterns of elite players assessing the impact of two distinct SSG tactical-conditioning sessions. The data demonstrated a greater TDR and HSR in the extensive compared to intensive SSGs. There was no meaningful difference between intensive and extensive SSGs for ACCs and DECs. On the other hand, a higher perceived intensity (session-RPE) was observed for intensive SSGs.

The present data are novel and add new insights into the internal and external loading of different formats of SSG tactical-conditioned training approaches. The findings from the present study could aid coaches who want to apply this approach using variations of common training modalities such as SSGs. Existing literature suggests that physical, technical and tactical metrics can be altered by varying the number of players, pitch dimensions, work to rest ratios, rules and coach encouragement (Dellal et al., 2008; Little and Williams, 2007; Rampinini et al., 2007). However, limited studies have been published that use the tactical objectives of the session as modulators of ETLs and ITLs. Interestingly, the present data demonstrate that coaches should consider not only the usual variables (rules, the number of players, etc), but also the tactical objectives of SSGs in relation to exercise bout duration and rest intervals.

Knowledge regarding the load experienced by elite soccer players performing SSG-tactical-conditioned training approaches is still scarce. In the present study, it was possible to examine the actual ETL and ITL for different types of SSGs, notably, intensive and extensive SSGs, using this approach. Data demonstrated a higher TDR and HSR for extensive SSGs compared to intensive SSGs. The prescription of this type of SSG was characterized by higher exercise bout duration and a greater number of players in comparison to intensive SSGs, while focusing on offensive/defensive coverage, unit training, pressure match-play situations, and delay training situations (for defence purposes). This suggests that this type of a session (extensive SSG) should be incorporated within periodization training, allowing players to be physically overloaded prior to the build-up for competition. This type of work could be used for developing tactical and technical elements while preparing players for match-play, during the early preseason conditioning phase. Alternatively, the current findings also suggest that when a coach wants to reduce the training distance, high-speed distance and impacts, it would be prudent to use an intensive SSG format. As accelerations and decelerations were not different between the different types of SSGs, this strategy might be useful as it may maintain these capabilities, while reducing fatigue levels associated with a greater training distance.

Interestingly, Buchheit et al. (2018) examined various neuromuscular indices using accelerometery during three typical soccer conditioned sessions (i.e. strength, endurance and speed), and reported that the assessed training sessions, performed in a horizontal alternation program were associated with limited neuromuscular fatigue, and that changes in neuromuscular performance and propulsion efficiency were session dependent. Considering the current findings and those of Buchheit et al. (2018), it is reasonable to infer that the loading of training sessions in both studies might be optimal to minimize fatigue accumulation throughout the microcycle. However, these results also suggest that greater training loads may need to be applied to generate optimal adaptation in elite players, and consequently, generating higher acute fatigue. These observations might guide practitioners and coaching staff in decision-making related to varying training loads and objectives.

It seems reasonable to assume that there would be numerous limitations when using periodization SSG approaches during the preseason. Therefore, team or isolated position-specific training drills should be incorporated together with SSG formats, to meet the demands of the modern game, thus allowing the translation of discrete technical sequences, angles of turns, and tactical actions (*Bradley* et al., 2018), while increasing the intense activity patterns that might not be addressed during tactical SSGs, such as longer sprints, accelerations, and positional specific demands.

Nevertheless, despite the results from the TDR and HSR in the present study, with a higher demand for the extensive SSGs, a higher session-RPE (perceived intensity) and ITL was observed for intensive SSGs. These findings corroborate the concept from Vanrenterghem et al. (2017), who proposed the adoption of a theoretical framework in which physiological and biomechanical loads should be separated in order to distinguish the ETL and ITL, as well as the load-adaptation pathways. The current results demonstrate that differences exist in the perceived intensity (psychophysiological measure) and the ETL of a particular training session, reinforcing the distinct load-adaptations pathways generated from the training sessions adopted in the present investigation.

## Conclusions

In summary, the results of the present study showed that tactical-conditioning SSG sessions promoted a different pattern of ETL variables, with a greater TDR and HSR magnitude for the extensive SSGs. Nevertheless, a higher session-RPE and ITL were observed for intensive SSGs. These results have consequences for practitioners and researchers for planning and designing experiments on training periodization and training load monitoring. The present results suggest that soccer coaches should consider the periodization applied to tactical-conditioning SSGs as a viable approach to impose specific ETLs and ITLs on soccer players. Such an approach could be used due to its specificity, time-efficiency and efficacy. The findings also suggest that when coaches aim to reduce the ETL associated to running distance during the preparation phase, they should use an intensive SSG session. Additionally, the results indicate that soccer coaches should implement a multi-dimensional training monitoring program, integrating ETL and ITL variables to better understand the training process.

## References

[j_hukin-2022-0083_ref_002] Ade J. D., Harley J. A., Bradley P. S. (2014). Physiological response, time-motion characteristics, and reproducibility of various speed-endurance drills in elite youth soccer players: small-sided games versus generic running. International Journal Sports Physiology and Performance.

[j_hukin-2022-0083_ref_003] Aguiar M., Botelho G., Lago C., Maças V., Sampaio J. (2012). A review on the effects of soccer small-sided games. Journal of Human Kinetics.

[j_hukin-2022-0083_ref_004] Aguiar M. V., Botelho G. M., Gonçalves B. S., Sampaio J. E. (2013). Physiological responses and activity profiles of football small-sided games. Journal of Strength and Conditioning Research.

[j_hukin-2022-0083_ref_005] Batterham A. M., Hopkins W. G. (2006). Making meaningful inferences about magnitudes. International Journal Sports Physiology and Performance.

[j_hukin-2022-0083_ref_006] Bradley P. S., Di Mascio M., Mohr M., Fransson D., Wells C., Moreira A., Castellano J., Gomez A., Ade J. D. (2018). Can modern football match demands be translated into novel training and testing modes?. Aspetar Sport Medicine Journal.

[j_hukin-2022-0083_ref_007] Buchheit M., Lacome M., Cholley Y., Simpson B. M. (2018). Neuromuscular responses to conditioned soccer sessions assessed via GPS-embedded accelerometers: insights into tactical periodization. International Journal of Sports Physiology and Performance.

[j_hukin-2022-0083_ref_008] Cohen J. (1988). Statistical Power Analysis for the Behavioural Sciences.

[j_hukin-2022-0083_ref_009] Dellal A., Chamari K., Pintus A., Girard O., Cotte T., Keller D. (2008). Heart rate responses during small-sided games and short intermittent running training in elite soccer players: a comparative study. Journal of Strength and Conditioning Research.

[j_hukin-2022-0083_ref_010] Foster C. (1998). Monitoring training in athletes with reference to overtraining syndrome. Medicine and Science in Sports and Exercise.

[j_hukin-2022-0083_ref_011] Gréhaigne J. F., Godbout P. (1995). Tactical knowledge in team sports from a constructivist and cognitivist perspective. Quest.

[j_hukin-2022-0083_ref_012] Halouani J., Chtourou H., Gabbett T., Chaouachi A., Chamari K. (2014). Small-sided games in team sports training: a brief review. Journal of Strength and Conditioning Research.

[j_hukin-2022-0083_ref_013] Impellizzeri F. M., Rampinini E., Coutts A. J., Sassi A., Marcora S. M. (2004). Use of RPE-based training load in soccer. Medicine and Science in Sports and Exercise.

[j_hukin-2022-0083_ref_014] Johnston R. J., Watsford M. L., Kelly S. J., Pine M. J., Spurrs R. W. (2014). Validity and interunit reliability of 10 Hz and 15 Hz GPS units for assessing athlete movement demands. Journal of Strength and Conditioning Research.

[j_hukin-2022-0083_ref_015] Little T., Williams A. G. (2007). Measures of exercise intensity during soccer training drills with professional soccer players. Journal of Strength and Conditioning Research.

[j_hukin-2022-0083_ref_016] Martín-García A., Gómez Díaz A., Bradley P. S., Morera F., Casamichana D. (2018). Quantification of a professional football team's external load using a microcycle structure. Journal of Strength and Conditioning Research.

[j_hukin-2022-0083_ref_017] Moreira A., Aoki M.S., Carling C., Rodrigues Lopes R. A., Arruda A. F. S., Lima M., Correa U. C., Bradley P.S. (2016). Temporal changes in technical and physical performances during a small-sided game in elite youth soccer players. Asian Journal of Sports Medicine.

[j_hukin-2022-0083_ref_018] Moreira A., Massa M., Thiengo C. R., Lopes R. A. R., Lima M. R., Vaeyens R., Barbosa W. P., Aoki M. S. (2017). Is the technical performance of young soccer players influenced by hormonal status, sexual maturity, anthropometric profile, and physical performance?. Biology of Sport.

[j_hukin-2022-0083_ref_019] Rampinini E., Impellizzeri F., Castagna C., Abt G., Chamari K., Sassi A., Marcora S. (2007). Factors influencing physiological responses to small-sided soccer games. Journal of Sports Science.

[j_hukin-2022-0083_ref_020] Sangnier S., Cotte T., Brachet O., Coquart J., Tourny C. (2019). Planning training workload in football using small-sided games' density. Journal of Strength and Conditioning Research.

[j_hukin-2022-0083_ref_021] Rodrigues Lopes R. A., Aoki M. S., Carling C., Vaz Ronque E. R., Moreira A. (2022). Do changes in fitness status, testosterone concentration, and anthropometric characteristics across a 16-month training period influence technical performance of youth soccer players during small-sided games?. Journal of Strength and Conditioning Research.

[j_hukin-2022-0083_ref_022] Teoldo I., Garganta J., Greco P. J., Mesquita I. (2009). Tactical principles of soccer game: concepts and application. Motriz.

[j_hukin-2022-0083_ref_023] Vanrenterghem J., Nedergaard N. J., Robinson M. A., Drust B. (2017). Training load monitoring in team sports: a novel framework separating physiological and biomechanical load-adaptation pathways. Sports Medicine.

[j_hukin-2022-0083_ref_024] Zanetti V., Carling C., Aoki M. S., Bradley P. S., Moreira A. (2021). Are there differences in elite youth soccer player work rate profiles in congested vs. regular match schedules? Journal of Strength and Conditioning Research.

